# Optimizing Infill Drilling Decisions Using Multi-Armed Bandits: Application in a Long-Term, Multi-Element Stockpile

**DOI:** 10.1007/s11004-017-9695-9

**Published:** 2017-07-17

**Authors:** Rein Dirkx, Roussos Dimitrakopoulos

**Affiliations:** COSMO–Stochastic Mine Planning Laboratoy, Department of Mining and Materials Engineering, McGill University, FDA Building, 3450 University Street, Montreal, QC H3A 0E8 Canada

**Keywords:** Infill drilling, Value of additional information, Multi-armed bandits, Multi-element stockpiles, Stochastic simulation

## Abstract

Mining operations face a decision regarding additional drilling several times during their lifetime. The two questions that always arise upon making this decision are whether more drilling is required and, if so, where the additional drill holes should be located. The method presented in this paper addresses both of these questions through an optimization in a multi-armed bandit (MAB) framework. The MAB optimizes the best infill drilling pattern while taking geological uncertainty into account by using multiple conditional simulations for the deposit under consideration. The proposed method is applied to a long-term, multi-element stockpile, which is a part of a gold mining complex. The stockpiles in this mining complex are of particular interest due to difficult-to-meet blending requirements. In several mining periods grade targets of deleterious elements at the processing plant can only be met by using high amounts of stockpiled material. The best pattern is defined in terms of causing the most material type changes for the blocks in the stockpile. Material type changes are the driver for changes in the extraction sequence, which ultimately defines the value of a mining operation. The results of the proposed method demonstrate its practical aspects and its effectiveness towards the optimization of infill drilling schemes.

## Introduction

Optimizing an infill drilling pattern can be linked to a more general problem present in many different applications such as stock markets, research effort direction, project development, and others. The more common term for this problem used across these various industries is the valuation of future information (under uncertainty). In terms of the economic utility theory, Schlee (1991) proves that perfect information always has a positive value. However, the requirement of a payment to retrieve this information might render the total value negative, causing a need to valuate this future information properly. In the mineral industry, Chorn and Carr (1999) assess the value of future information for decisions on additional capital investments, and Prange et al. (2008) valuate information gathering campaigns on the sealing capacity of a fault system.

In the aforementioned works, the value of future information is a monetary value. This is hard to quantify for a mineral deposit. Boucher et al. (2005) indirectly do this via a misclassification cost of material. This misclassification refers to ore versus waste based on a fixed cut-off grade and one processing stream. However, this is usually not the case in a mining complex. Menabde et al. (2007) show that it is optimal to use a variable cut-off grade that comes directly from the schedule optimization. This makes it impossible to evaluate misclassification errors without rescheduling the deposit. This effect is strengthened if multiple processing streams are considered in combination with the blending of material from different sources, as in the application presented below.

Barnes (1989), Diehl and David (1982), Gershon et al. (1988), Delmelle and Goovaerts (2009) and others explore infill drilling optimization by focusing on minimizing the kriging variance or trying to locate zones of high kriging variance as prime spots for additional drilling or alternative approaches . A flaw in using kriging variance as a measure of variability is that it only captures the geometric part of the uncertainty for a drilled deposit and does not take into account the variability of grades (Goovaerts 1997; Journel and Kyriakidis 2004; Rossi and Deutsch 2014). Ravenscroft (1992) shows that working with geological simulations is the best way to represent variability in a deposit and that estimated orebody models give a flawed representation of the true variability in a deposit. Therefore, simulations are used in this approach to infill drilling optimization and the assessment of additional patterns under geological grade uncertainty. Goria et al. (2001) also propose a method based on conditional simulations to assess the value of additional drill holes. An important conclusion from this work is that additional drilling does not necessarily reduce the variability in the deposit. Boucher et al. (2005) propose a method for the optimization of infill drilling that also makes use of simulated orebody models and compares the cost of drilling additional drill holes to the misclassification cost of the material. The aforementioned works inform the additional drill holes with a simulated grade, drawn from simulations based on the initial exploration data. This is used as a possibly ”true” grade in the added value calculations. A similar approach is taken here.

The major contribution of the proposed method is the use of multi-armed bandits (MAB) for the optimization. Their benefit is that they provide an elegant solution to the requirement of testing every single simulation as possibly ”true” representation multiple times. The MAB framework origins in the world of casinos. The initial problem comes from playing a row of slot machines (commonly referred to as an armed bandit) in a certain sequence in order to maximize the total long-term reward from playing these machines. This provides an analogy with the well-known exploration versus exploitation trade-off present in many other applications such as clinical trials and internet advertisements. The exploration versus exploitation trade-off in infill drilling exists in the availability of many possible locations for additional drill holes with each an unknown outcome. The proposed method applies the MAB framework to find the best infill drilling pattern from a predefined set of patterns.

This paper considers additional information for a mineral deposit to be valuable if the extraction schedule is influenced. Differences in extraction schedules are often caused by a change in material types of various blocks. Therefore, value is linked to material type changes, the main driver of schedule changes. The definition of the best pattern is the one that adds the most value to the knowledge. According to this paper’s definition of value, the best infill drilling pattern is the one that causes the most material type changes, implicitly linked to schedule changes. The case study presented herein requires simulations of a multi-element stockpile. This requires a simulation technique capable of dealing with multiple spatially correlated variables. The simulations throughout the remainder of this work are all generated using minimum/maximum autocorrelation factors (MAF) (Switzer and Green 1984). Desbarats and Dimitrakopoulos (2000) describe the MAF-technique in a mineral science context by applying it to the simulation of pore-size distribution. Boucher and Dimitrakopoulos (2009) apply it directly to the block support level by combining it with direct block simulation (Godoy 2002); it is this direct block method that is used below.

The three main contributions illustrated above show that this method distinguishes itself from the past work through all three of them. Firstly, a new framework in infill drilling optimization, the MAB, takes patterns and the interactions between the constituting drill holes into account. Secondly, an unused definition of added value in the form of material types changes which effectively have the possibility to change mining schedules, the real value drivers of mining operations. Finally, the applicability to a multi-element deposit, by using MAF simulation, which stresses the importance of a proper value definition even more.

In the following sections, first the details of the proposed method are addressed. Once the method is explained, the need for additional information for stockpiles is illustrated and an application of the infill drilling optimization for a long-term, multi-element stockpile is shown. Finally, a conclusion and recommendations for future work are given.

## Method

### Multi-Armed Bandits

The classic and simplest MAB framework follows the four basic rules formulated below as in Mahajan and Teneketzis (2008). The state for an arm is the internal time, which is equal to the number of times the arm has been played.

1. Only one arm is played at each time-step. The reward from this play is uncontrolled, meaning that the operator only controls what arm is played not how it is played.
2. Not played arms remain frozen, meaning they do not change state.
3. A frozen arm will not give a reward.
4. All arms are independent.

There are many variants of MAB problems where one or more of these rules are omitted or altered, which makes them considerably more complicated to understand and solve. Therefore, the focus of the following sections will only be on these simple bandits, as they are sufficient to address infill drilling.

The objective function of the MAB problem, *k* with arms, is given in Eq. (1). The same formulation as in Mahajan and Teneketzis (2008) is used

1 $$\begin{aligned} J^{\gamma }={\mathbb {E}}\left[ {\mathop {\sum }\nolimits _{t=0}^\infty \beta ^{t}\mathop {\sum }\nolimits _{i=1}^k R_i({X_i({N_i(t)}), U_i(t)})|Z(0)}\right] , \end{aligned}$$

with, $J^{\gamma }$, the objective function value under policy $\gamma$, defined by $U_i(t)$. The discount factor with time *t*, $\beta ^{t}$, reflects that earlier rewards are higher valued than later rewards, like in a Net Present Value (NPV) calculation. The time *t* is a measure for the number of iterations/plays. The reward of arm $i, \,R_i({X_i( {N_i(t)}),U_i(t)})$, depends on $X_i ({N_i(t)})$, the state of arm *i* at time *t*, which depends on $N_i(t)$, the local time of arm *i* at time *t*; the number of times arm *i* has been played before time *t*. The policy decision, $U_i(t)$, determines whether or not arm *i* is played at time *t*. Finally, *Z*(0), the initial state of all arms *i*. The objective function value is calculated by taking the expected value over the summations over time and the number of arms conditional to the initial states of all arms. The goal is to find the optimal scheduling policy that determines the values of $U_{i}(t)$ for every arm *i* and time *t* in such a way that the total expected reward is maximized. The MAB problem can also be used to find the best performing arm, instead of a schedule of when each arm should be played. The best arm is just the only one that occurs in the schedule after some initial exploration of the other arms.

### Thompson Sampling

The optimization algorithm for the MAB is a heuristic algorithm called Thompson sampling (Thompson 1933), a popular heuristic in artificial intelligence and for solving MAB problems. The general idea of Thompson sampling is: always play the arm with the highest likelihood of providing the highest reward. According to this rule, it is always possible to explore arms that have not performed as well in the previous iterations, but it is most likely that the best performing arms are exploited. This gives a natural approach to the exploration versus exploitation trade-off in MAB. Important to remark here is that infill drilling is never referred to as sampling to avoid confusion with the solution algorithm, Thompson sampling.

Thompson sampling is chosen because it has shown excellent performance for many problem instances (Agrawal and Goyal 2012; Chapelle and Li 2011; May and Leslie 2011; Scott 2010), and it requires weaker assumptions on the knowledge of the prior reward distributions of the arms. This is especially beneficial because it is not known how the material type changes due to a drilling pattern are distributed, hence, the true reward distributions are unknown. This is common in many applications of MABs and is usually overcome in Thompson sampling by assuming a uniform prior over [0,1] for each arm. The prior is represented as a beta-distribution with parameters $\upalpha = 1$ and $\upbeta = 1$ (B[1,1]). This leads to a pure exploration phase at the beginning of the algorithm because all distributions are identical. The algorithm gradually evolves to a pure exploitation phase of the highest reward-giving arm at convergence. The beta-distribution is chosen to represent the reward distribution of the arms because it is easy to interpret for the rewards and easy to update during the Thompson sampling algorithm as well.

The distribution update depends on the reward of the pull. Traditionally, the distribution is updated according to whether a pull is a success (reward is 1) or a failure (reward is 0). If it is a success, the posterior of arm *i* is updated to $\hbox {B}_{\mathrm{i}}(\alpha _{t} + 1,\, \beta _{t})$ at iteration *t*; otherwise, it is updated to $\hbox {B}_{\mathrm{i}}(\alpha _{t}$, $\beta _{t} + 1$) at iteration $t,\, \alpha _{t}$ and $\beta _{t}$, which are the parameters of the beta-distribution at iteration *t* updated for all previous iterations. Because the reward is defined as the percentage of blocks that change their material, it is not binary, but it is defined on [0,1]. This adds a slight complication to the updating of the distribution. This complication is resolved by first performing a Bernoulli trial with the reward between 0 and 1 as the success rate. The binary result of the Bernoulli trial is then used to update the distribution in the same way as explained above. Pseudo-code for the Thompson sampling algorithm with Bernoulli trial updates is provided in Algorithm 1. The parameters of the beta-distribution follow the same definition as above. The parameter T in the algorithm is a generic parameter used to denote the stopping time of the algorithm.

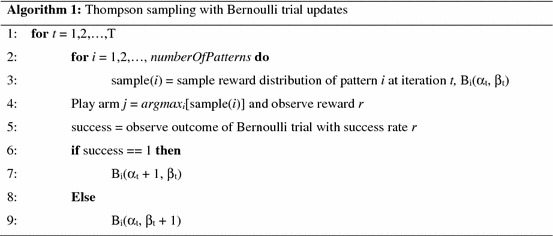



The Thompson sampling algorithm is proven to converge for the MAB problem by Agrawal and Goyal (2012) and May et al. (2012), but it has no predefined termination moment. Therefore, it is required to define a convergence criterion that works for the application in consideration. In this paper, a simple criterion is defined, which has shown satisfactory results. That is, if in the last 20% of iterations one arm is selected to be played more than 90% of the iterations, then this is the best arm. Important to note here is that this criterion is not checked for the first iterations (e.g. 100) to allow for some initial exploration.

### Algorithm

**Fig. 1:**
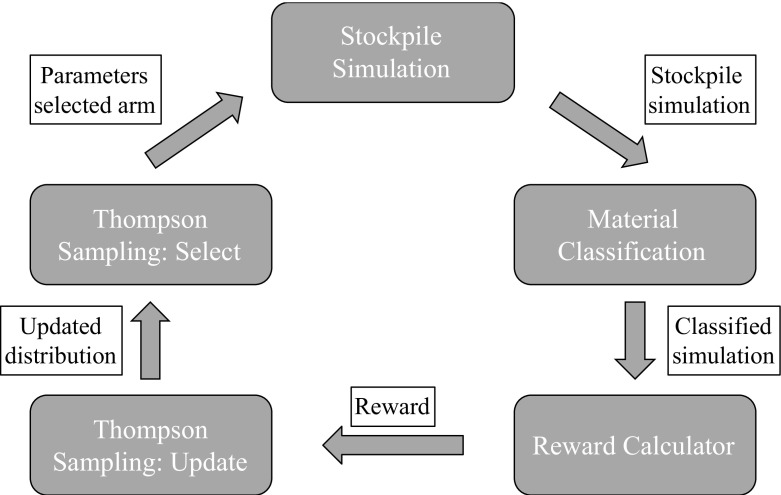
High-level schematic representation of the MAB optimization algorithm

The proposed method selects the best infill drilling pattern from a predefined set of patterns. The representation of each pattern for infill drilling will be called an arm in the MAB framework. These arms will be played/pulled (these terms will be used interchangeably throughout the remainder of the text). When an arm is played, the algorithm assesses the value for the pattern associated with that arm is set in motion. The steps in this value assessment are the following (Fig. 1 demonstrates the general flow of the algorithm):

1. The grades in the drill holes of the selected pattern are drawn from an initial simulation, which is set to be the possibly ‘true’ representation of the deposit.
2. The drill holes of the pattern linked to the pulled arm are used as additional data to the initial exploration data for the re-simulation of the deposit.
3. After the re-simulation with additional data, the blocks of the re-simulation are classified according to a material classification guide.
4. The reward or contribution from pulling that arm is defined as the percentage of blocks that have changed material classification in the re-simulation compared to the possibly ‘true’ deposit. Optimizing for this reward is in line with the definition of the best pattern given above.
5. The Thompson sampling solution algorithm updates the current arm and the next arm is selected.

After convergence of the algorithm, the whole procedure is repeated for other simulations of the deposit as a possibly ”true” deposit in order to test the sensitivity of the method to this choice. The best pattern is then selected over the results of all of these possibly ”true” deposits. By considering multiple simulations as possibly ”true” deposits, the method is able to give an assessment of the performance of all patterns under geological uncertainty, which leads to the quantification of the upside potential and downside risk for each pattern. All of the patterns in one MAB set-up are comparable in the sense that they belong to what is called the same budget class, which means that they all have the same amount of drill holes and thus represent a similar drilling cost. The procedure is repeated for different budget classes of patterns, where each budget class represents patterns with a different number of holes than in the other classes. A separate MAB is required to optimize within each budget class, as the rewards of patterns with more holes cannot be directly compared to the rewards of patterns with fewer holes.

## Case Study–A Long-term, Multi-element Stockpile

The case study presented below illustrates the optimization of the infill drilling decision for a long-term, multi-element stockpile of a gold mining complex in Nevada, USA. This complex consists of two open pit mines, an underground mine and several stockpiles. The downstream processing includes an autoclave, an oxide mill and multiple heap leaches. Deleterious compounds are co-simulated with the gold grade because of strict grade requirements on them to guarantee efficient processing recoveries. Knowledge of the grade of these compounds can assure blending of ore from different sources to meet the constraints at the processing facilities. The compounds being considered are sulphide sulphur, organic carbon and carbonate, next to gold as the paying metal. This case study focuses on one stockpile in the mining complex.

For this case study, three classes of patterns are considered, each corresponding to a different budget. The first budget class has patterns with five holes, the next class has patterns with ten holes, and the last class has patterns with 15 holes. Eight patterns, based on the ideas provided to us by the mine site, are considered for the optimization in each class. For each class, the procedure is repeated for 20 simulations as possibly ”true” stockpiles to guarantee that the results are independent from the selected possibly ”true” stockpile. This also assesses the performance of the patterns under geological uncertainty. A validation step tests all patterns with simulations not used in the optimization procedure and shows that the results are independent of the simulations employed.

### Stockpile Simulations

As mentioned in the introduction, the MAF technique with direct block simulation (Boucher and Dimitrakopoulos 2009) is used to generate the stockpile simulations because there are four correlated compounds of interest. The MAF technique decorrelates the variables via two eigenvalue decompositions, first on the covariance matrix of the original data and second on the covariance matrix at a chosen lag. The resulting decorrelated variables can be simulated independently and maintain their spatial correlations after back transformation. The first element is gold (Au), which is the produced metal. Sulphide sulphur (SS), organic carbon (OC), and carbonate ($\mathrm {CO}_{3}$) are of importance for the working of the downstream processing facilities. The main constraints at the autoclave, where the sulphide material from the stockpile is processed, are a maximum organic carbon content and an upper and lower bound on the sulphide sulphur-carbonate ratio. Using the above mentioned method, 20 simulations are generated each containing 204 blocks within the shape of the stockpile. The block size is 30 $\times$ 30 $\times$ 20 ft. This is the same as in the mines feeding the stockpile and processing streams because the same mining selectivity is used for both the stockpiles and the mines.

**Fig. 2:**
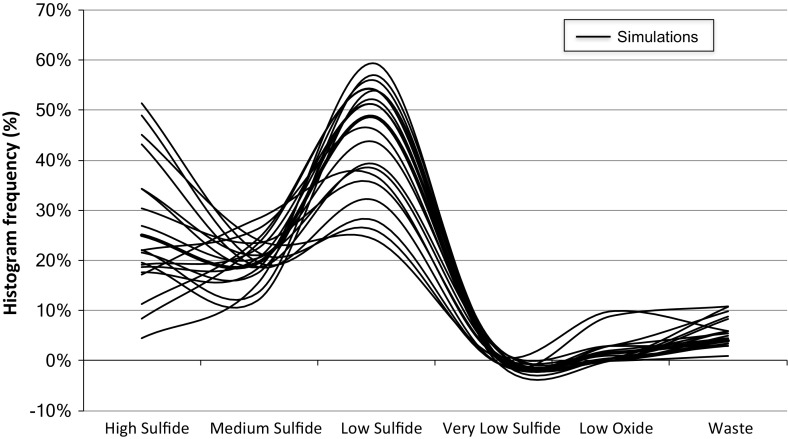
Histograms of the material variability at the block level over all 20 stockpile simulations

The prior drilling of the stockpile is done on a regular grid with a 40 ft. spacing. In total, there are 104 drill holes with one sample each. The height of the stockpile is 20 ft. The size is ±200000 m3, which corresponds to ±400 kton. Figure 2 shows the material variability over all 20 stockpile simulations at the block level. The material is classified based on the material classification guide provided by the mining complex. The distinction between the different sulphide materials (e.g. high sulphide versus medium sulphide) is made based on gold grade. The difference between oxide versus sulphide material comes not only from the gold grade, but also from the other compounds. The mining complex flags the stockpile under consideration as medium sulphide material, but Fig. 2 shows that in all simulations there is less than 30% medium sulphide material present. High and low sulphide material usually dominate the material distribution in the stockpile. These two material types also have a much higher variability in quantity over all simulations than the medium sulphide material. This can be in part explained by the fact that the gold grade criteria for the medium sulphide material are tighter than for the low sulphide or the high sulphide, which does not have a cap for gold grade. However, it still shows a high amount of misclassified material.

**Fig. 3:**
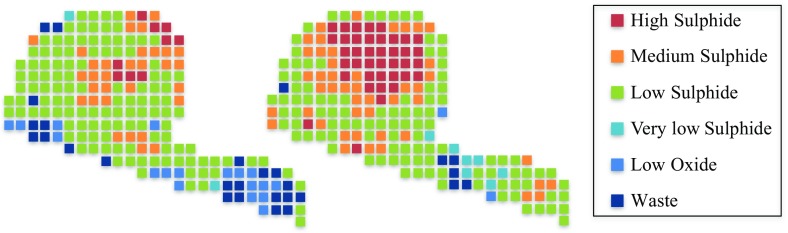
Spatial material variability of the stockpile simulations at the block level

**Fig. 4:**
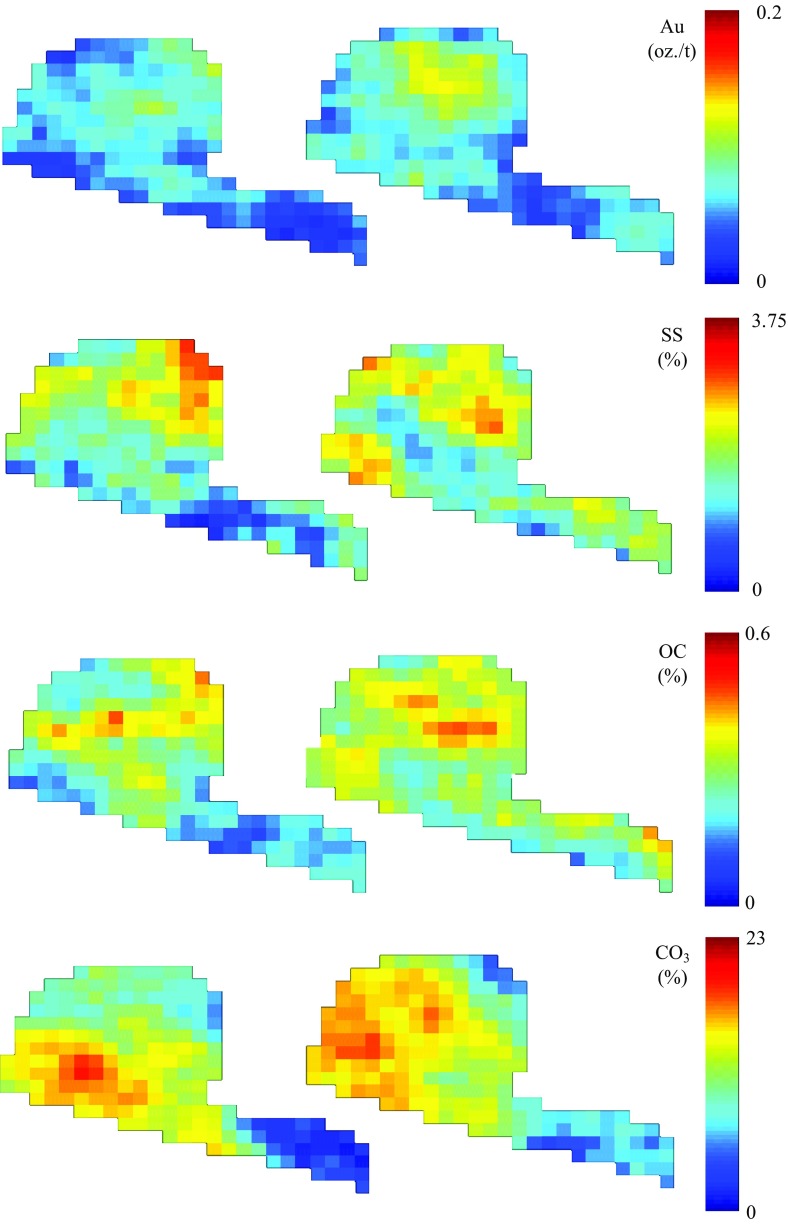
Gold, sulphide sulphur, organic carbon, and carbonate content variability in two simulations at the block level

Similar results can be seen from Fig. 3, which demonstrates the spatial material variability for two simulations on the block level. For this case study, the high and low sulphide materials that dominate the material types in the stockpile. Figure 3 also shows that there is a large amount of spatial material variability in the stockpiles within one simulation, but also from one simulation to another. This observation is one of the main motivations for the application of the proposed method to stockpiles. It shows that more information is required to assess the local scale variability in stockpiles. This is especially true for the stockpile in this mining complex because, in some periods, it is the main contributor to the processed blend at the autoclave. Figure 4 shows the spatial material variability in two simulations for the four variables of interest. All simulations show that the same main features are respected between two simulations, but also that there is local scale variability for the grade within each simulation. The gold simulations, at the top of Fig. 4, show the presence of high grades in the centre and toward the top of the ball shape on the left side of the stockpile. The high sulphide material zones shown in Fig. 3 are a result of these high gold grades.

### Tested patterns

**Fig. 5:**
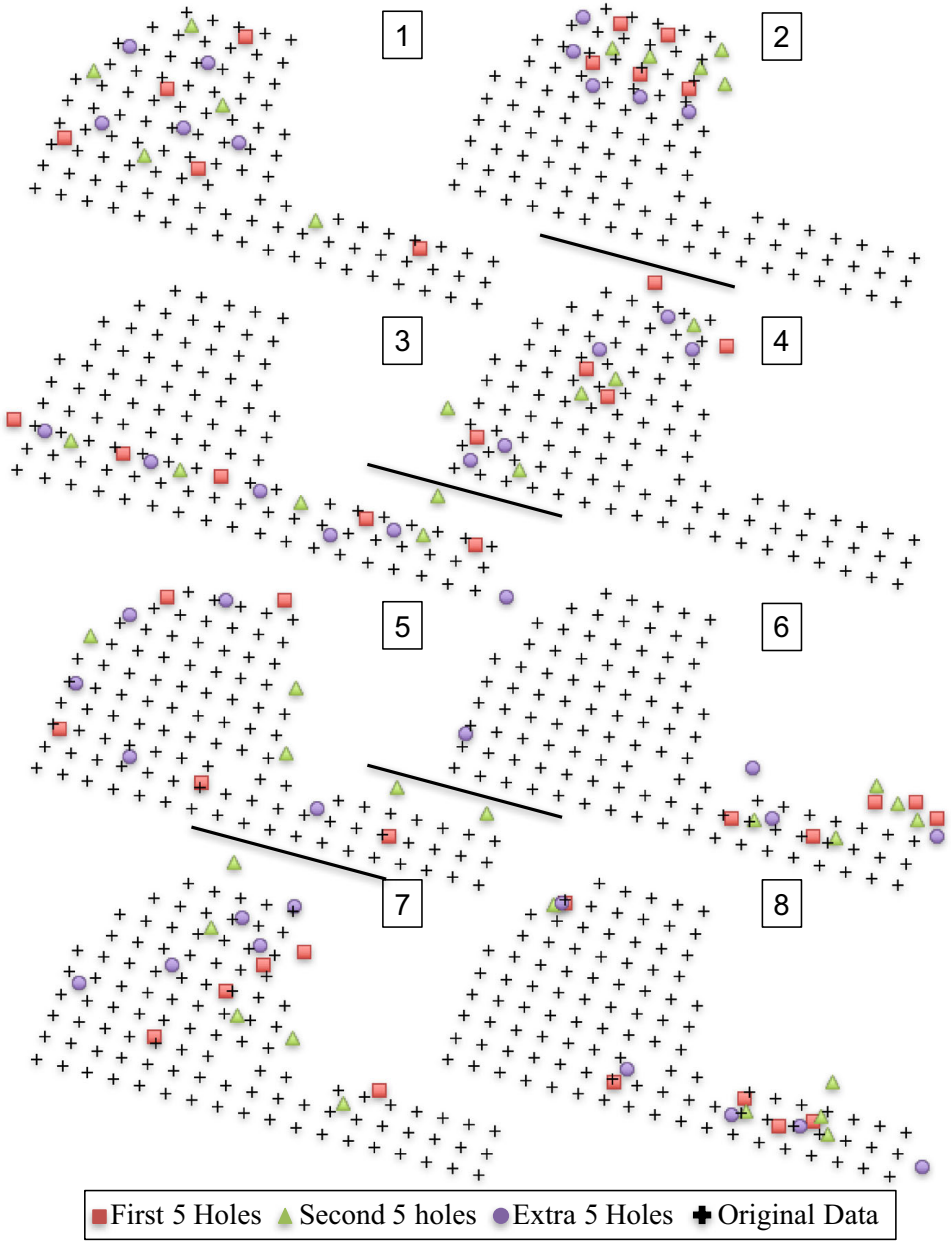
The eight patterns with 15 additional drill holes

Figure 5 shows the eight patterns that are considered in the optimization. The patterns are always shown with all 15 additional drill holes on top of the initial exploration drill holes which are represented as black crosses. The red squares (first five holes) represent the budget class with five holes per pattern. The red squares and the green triangles (second five holes) represent the budget class with ten holes per pattern. The patterns have the same numbering over the budget classes because they are based on the same idea for the location of the additional drill holes. The patterns are constructed based on ideas provided to us by the mine site.

Pattern 1 is constructed as an evenly spread pattern through the whole stockpile. Pattern 2 is constructed as a pattern that focuses on a high gold grade zone at the top of the stockpile. Pattern 3 is constructed to mimic the delineation of the stockpile on the road side. Pattern 4 is constructed to target some specific zones of high gold grade spread over the stockpile. Pattern 5 is constructed to delineate the borders of the stockpile more precisely. Pattern 6 is constructed based on the blocks (30 $\times$ 30 $\times$ 20 ft.) that have the highest coefficient of variation in gold grade over the 20 stockpile simulations. Pattern 7 is constructed by randomly selecting drill hole locations. Pattern 8 is constructed as a mix of points and blocks with a size of 10 $\times$ 10 $\times$ 10 ft. with the highest coefficient of variation over all 20 simulations. It was required to mix these two scales to look at the highest coefficients of variation because the 15 points with the highest coefficients of variation are located right next to another. A pattern like that would not make sense. To add some diversity in the locations of the additional drill holes the blocks of size 10 $\times$ 10 $\times$ 10 ft. with the highest coefficient of variation over all 20 simulations are considered as well.

### Case Study Results

**Fig. 6:**
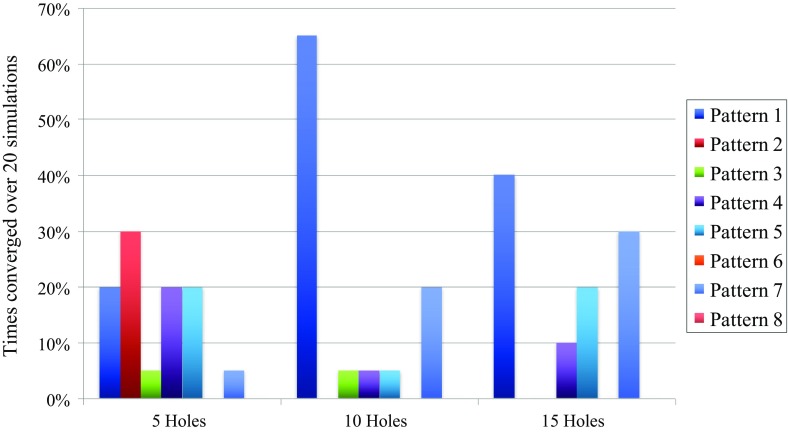
Convergence results over all 20 simulations for all patterns per budget class

Figure 6 shows the convergence results for the algorithm over all simulations for every pattern in each budget class. Convergence results show how often the algorithm has converged on that specific pattern in that budget class for the 20 simulations as a possibly ”true” deposit. The pattern that has been converged upon the most is called the ”winner” of that budget class. For the ten- and 15-hole budget classes, there is always a clear winner in pattern 1. However, for both budget classes, it has to be noted that pattern 7 also shows comparable performance, especially for the 15-hole budget class. Identifying a winner for the five-hole budget class is less obvious. Pattern 2 has 30% convergence, but patterns 1, 4, and 5 each have 20% too. This is only a small difference and no clear decision on the winner can be made from this.

**Fig. 7:**
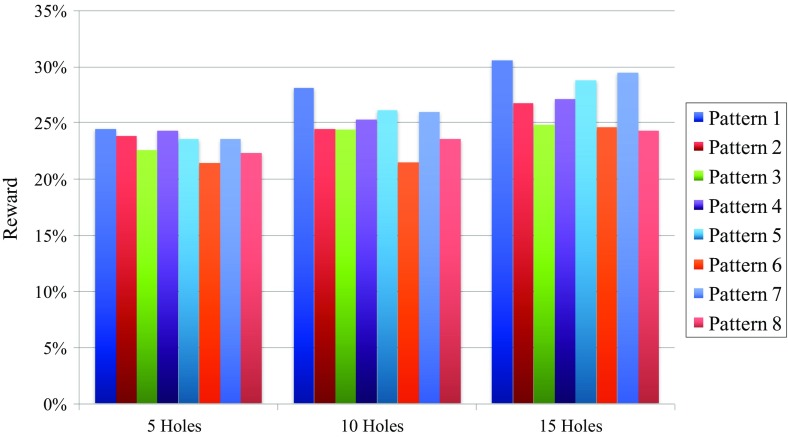
Average reward over all 20 simulations for all patterns per budget class

Figure 7 shows the average reward over all 20 simulations for each pattern in each budget class. For the ten- and 15-hole budget classes, it is pattern 1 that has the highest average reward, as was expected from the convergence results in Fig. 6. The link between these figures is apparent. However, the results for the five-hole budget class are not as expected in the sense that it is pattern 1 and not pattern 2 that has the highest average reward. This can be explained by pattern 2 strongly outperforming pattern 1 for some simulations as the possibly ”true” stockpile, while in others pattern 2’s performance is much worse than pattern 1’s. Therefore, it can be concluded that pattern 1 is more robust against geological uncertainty than pattern 2. Figure 7 corroborates the observation of no clear winner for the five-hole budget class by demonstrating that the average rewards of all patterns are very close to each other. It is hard for the MAB to distinguish between patterns with such similar rewards.

Table 1 shows the running times, the number of iterations, the number of times the algorithm did not converge, and the patterns causing the non-convergence, for the optimization of each budget class over all simulations. The differences between the running times for the different budget classes observed in Table 1 can also be explained by the observations in Figs. 6, 7. Because the five-hole patterns yield similar average rewards, the algorithm has difficulties finding the winner and, therefore, it requires more iterations, and thus more time, to evaluate the patterns over all simulations. The five-hole budget class requires almost three times more time than the 15-hole budget class and almost twice the time than the ten-hole budget class. The number of iterations to evaluate all patterns within one budget class for all simulations is very high. In fact, it is higher than the brute force approach, which tests every pattern 20 times for each simulation and then takes the average. This brute force tactic would result in 8 $\times$ 20 $\times$ 20 = 3200 iterations to evaluate each budget class. The numbers higher than 3200 in Table 1 come from a few simulations with hard convergence problems that add a lot of iterations to the total count. These also explain the high running times.

**Table 1:** Overview of the statistics of the algorithm for each budget class

Budget Class	Running time	Iterations	Not converged	Non-converging patterns
5 Holes	24 h 24 min 46 s	25,465	3	**2** vs. 6, 1 vs. **2**, 1 vs. **4**
10 Holes	13 h 9 min 31 s	13,085	4	1 vs. **3**, **1** vs. 2, **1** vs. 2, **1** vs. 4
15 Holes	**8 h 41 min 31 s**	**9,378**	**2**	**1** vs. 2, 3 vs. **4**

The last column shows the patterns that caused the non-convergence. The pattern that has the highest average reward in case of a tie is marked in bold and is attributed the convergence

The number of non-converging simulations is low for every budget class. To get it to zero, unnecessary large numbers of iterations are required because it is always possible that two patterns perform very similarly for a certain simulation. Therefore, it is not really seen as a problem because the algorithm still identifies the two patterns that perform equally well in these cases. As a tiebreaker, the pattern with the highest average reward is attributed the convergence. A summary of which patterns caused the non-convergences for each budget class is also presented in Table 1 in the last column. A small sidenote is that a different set-up is used for the five-hole pattern optimization. This set-up allows for 5000 instead of 2000 iterations as the maximum iterations without convergence. Another addition to this set-up is a forced exploration-phase at the start of the algorithm forcing each pattern to be played 20 times before the algorithm starts doing its normal steps. Both measures are taken to overcome convergence issues observed in initial tests.

**Fig. 8:**
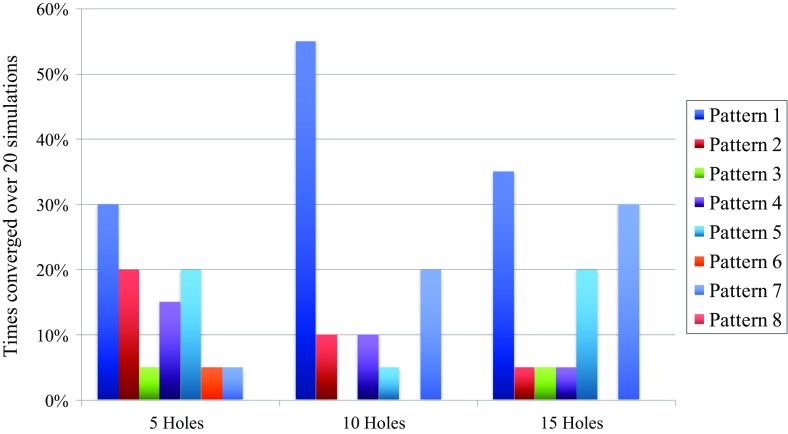
Convergence results over all 20 simulations for all patterns per budget class when the ties presented in Table 1 are broken in the opposite way

Figure 8 shows the convergence results if the ties in the non-convergence cases of Table 1 are broken in the opposite way, the average rewards for each pattern are obviously unaltered in this case. For the five-hole budget class, pattern 1 gains 10% and pattern 2 loses 10%, making pattern 1 the most converged upon as indicated by the highest average reward in Fig. 7. Pattern 4 loses 5% and pattern 6 gains 5%, but this does not alter the conclusions made above. For the ten-hole budget class, pattern 1 loses 15% but also gains 5%, this means a net-loss of 10%. Pattern 2 gains 10%, pattern 3 loses 5% and pattern 4 gains 5%. All these changes do not alter the conclusions for the ten-hole budget class; it is still pattern 1 that performs the best. For the 15-hole budget class, pattern 1 and 4 each loose 5% and patterns 2 and 3 each gain 5%. This does not alter the conclusions either. However, the difference between the convergence percentage of patterns 1 and 7 is only 5% in this case, making pattern 1 a less clear winner in terms of convergence percentage. The difference in average reward is still sufficient to conclude that pattern 1 is better than pattern 7 for the 15-hole budget class. As an intermediate conclusion from the graphs in Figs. 6, 7, and the discussion on the non-convergence, pattern 1 is the best performing pattern for each budget class. In addition to this, it is important to remark that the five-hole patterns might not be sufficient for this stockpile. This is concluded from the fact that their average rewards are all very similar no matter where the exact locations of the drill holes are.

**Fig. 9:**
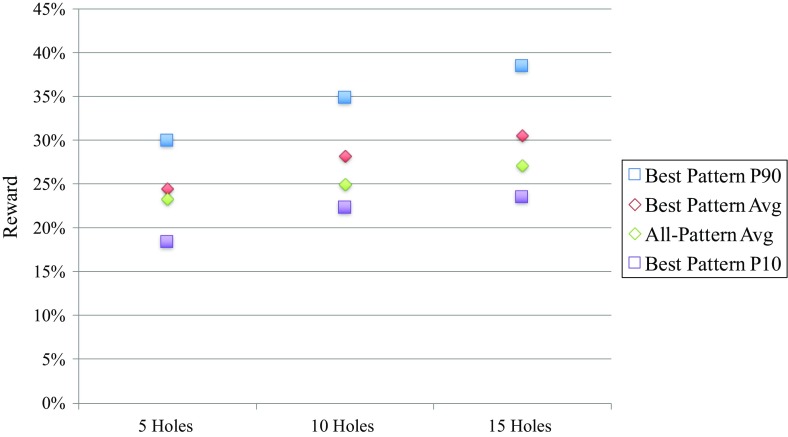
Profile of the best pattern per budget class versus the all-pattern average per budget class

Figure 9 compares the performance of the patterns over the budget classes. This graph demonstrates the profiles (P10-P50-P90) on the best pattern’s reward over all simulations compared to the average of the rewards of all patterns over all simulations. The tenth percentile (P10) represents the downside risk and the ninetieth percentile (P90) represents the upside potential of the best pattern under geological uncertainty. The five-hole best pattern average is lower than the all-pattern average for both other budget classes. Also, the downside risk is much higher and the upside potential is much lower. Therefore, in combination with the reasons mentioned above, it is not recommended to choose a five-hole pattern for infill drilling in this stockpile. Purely based on performance, pattern 1 with 15 holes is better than pattern 1 with ten holes. However, the extra five holes also cost more to drill. Another observation is that the downside risk for the 10- and 15-hole pattern 1 is almost equal. This means that they both guarantee similar results in the worst-case scenario. This is an extra argument in favour of pattern 1 with ten holes, especially if there are constraints on the budget that are more important than having the best possible knowledge.

### Validation of the Results

**Fig. 10:**
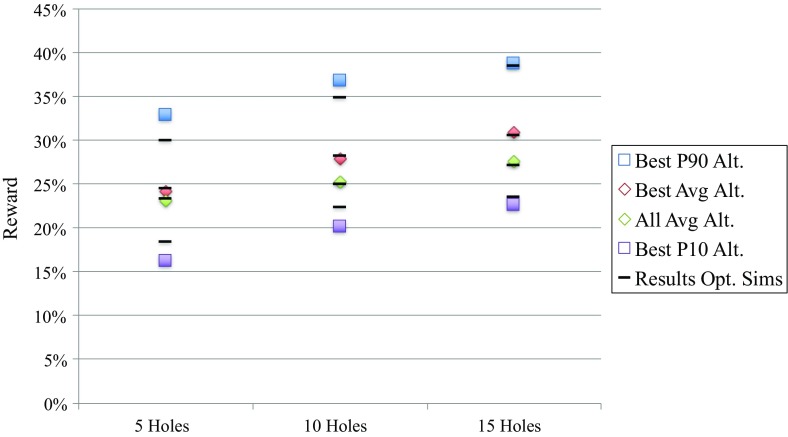
Profile of the best pattern per budget class versus the all-pattern average per budget class based on the reward from the alternate set of simulations presented by the *squares* and *diamonds*

As a validation step, the performance of the patterns is tested on an alternate set of 20 simulations, which are not used in the optimization process. Every pattern is tested 20 times for each simulation in the alternate set. One test is the same as the evaluation procedure in the MAB described above; the stockpile is re-simulated based on the pattern data from the possibly ”true” stockpile plus the original data and the reward is calculated. 20 tests for every simulation result in 400 rewards for every pattern in each budget class. The rewards are summarized by their profile based on the tenth and ninetieth percentiles as demonstrated in Fig. 10. This figure is the counterpart of Fig. 9. For ease of comparison, the results of Fig. 9 are summarized with the black lines in Fig. 10. The black lines are always very close to their counterpart from the tests on the alternate set of simulations. The results of Fig. 10 lead to the same conclusion as made for Fig. 9 above. The validation demonstrates that the proposed optimization method produces results independent of the simulations used.

## Conclusions

The paper presented above shows that the proposed MAB algorithm is able to select the best pattern within a set of patterns of the same budget and provides a decision tool to select the best pattern between sets of a different budget. Also, the case study demonstrates how the method successfully quantifies the influence of geological uncertainty on the performance of an infill drilling pattern. Additional to the performance of the proposed infill drilling optimization algorithm, it is demonstrated that stockpiles can be effectively simulated and that their local scale variability can be much higher than expected. The method is applied to a stockpile in this case study but its applicability is not limited to stockpiles. The proposed method can be used to optimize an infill drilling campaign for any deposit in any stage, as long as some prior information on the variables of interest is available.

The proposed approach could be improved by updating the original simulation after every play according to the infill drilling data, instead of performing a re-simulation at every iteration. Two methods that are suitable for this are conditional simulation by successive residuals (Jewbali and Dimitrakopoulos 2011) and ensemble Kalman filters (Benndorf 2015). Another improvement to the algorithm could be to let it choose the locations of the additional drill holes independently, rather than to constrain it to a set of predefined patterns.
